# Medical and Physical Intelligence

**Published:** 1801-06

**Authors:** 


### MEDICAL AND PHYSICAL INTELLIGENCE.

### [ FOREIGN AND DOMESTIC. ]

*On the illumination of rotten wood.*—The illumination of rotten wood has been of late a ſubject of enquiry and diſcuſſion amongſt naturaliſts. The late *Mr. Spallanzani* maintains, that there is a perfect analogy between the illumination of rotten wood and the artificial phoſphorus, and he imagines, that by the putrid fermentation, the hydrogen and the carbon of the wood come more eaſily in contact with the oxygen of the atmoſphere; by which combination, a ſlow combuſtion and illumination are produced, and he at the ſame time thinks, that this proceſs cannot proceed in the irreſpirable kinds of gaſſes. Rotten wood alſo, in which the neceſſary quantity of hydrogen and carbon is not at the ſame time diſengaged, does not obtain the property of illuminating. *Mr. Corradori*, however, objects to this theory, that a ſlow combuſtion cannot well take place, as the wood, at the time when it begins to illuminate, is moſtly deprived of its reſinous particles, containing, conſequently, but very little hydrogen and carbon; and it appears to him very probable, that the more it loſes of combuſtible matter, the more it obtains the property of illuminating. There is, in ſhort, he ſays, a very great difference between this natural and the artificial phoſphorus.—*Mr. Humboldt*, whoſe experiments we have already mentioned, concludes from them, that the illumination of rotten wood takes place only when it gets in contact with oxygen; and when it has loſt the property of emitting light in irreſpirable gaſſes, it recovers it again by expoſing it to oxygen gas. *Dr. Gaertner*, however, is of opinion, that, according to his experiments, a certain degree of humidity is always requiſite, and he thinks that oxygen gas is not quite neceſſary, though the illumination is increaſed by it. This phenomenon, however, being very different from all known proceſſes of combuſtion, where light is diſengaged, *Dr. G.* aſks, whether the illumination of the wood does not more agree with the animal proceſs of reſpiration than with a true combuſtion? or, whether it is produced by phoſphorus and carbon, in a proportion hitherto unknown? *Dr. G.* is, on the whole, inclined to think, that it is at preſent impoſſible to give a ſatisfactory explanation of the phenomena that occur in this proceſs. *Mr. Boeckmann* has made numerous experiments and obſervations on the illumination of rotten wood in different gaſſes and fluids, in order to throw ſome light on the ideas of the above naturaliſts. The reſults of theſe experiments differ in ſome points from what the experiments of thoſe gentlemen have ſhown, which circumſtance, however, *Mr. Boeckmann* attributes to the nature of rotten rotten wood, as a fubftance that is not always of the fame kind, and has not always an equal degree of putrefaction and humidity. It feems to differ likewife materially from the artificial phofphorus by the following diagnoftics.

1. It fhines in oxygen gas, at a very low temperature.

2. It emits light in all refpirable gaffes, at leaft for a fhort time.

3. In muriatic gas its light is fuddenly extinguifhed.

4. It fhines with a lefs degree in air rarified by the air pump.

5. According to the experiments of *Mr. Corradori,* it even fhines in the toricellian vacuum.

6. Its illumination is extinguifhed in oxygen gas as well as in other kinds of irrefpirable gaffes, when they are heated.

7. By its illumination in oxygen gas, carbonic gas is produced.

8. One may fuffer the rotten wood to be extinguifhed feveral times, one after another, in irrefpirable gaffes, without depriving them of the property of making a new piece of luminous wood fhine again for a fhort time.

9. Humidity greatly promotes the illumination, and feems even to be neceffary in producing it.

10. The rotten wood continues to fhine under water, oil and other fluids, and in fome of them its light is even increafed.

All this feems to fhow, that the extinction of the rotten wood in different media, does not immediately depend on a want of oxygen, but rather on a particular change to which the wood itfelf has been expofed.

---

*Dr. Kurzwig,* of Riga, relates the hiftory of a fuccefsful Cæfarean operation, by which a dead child was extracted. The pelvis of the woman, on whom the operation was performed, meafured only two inches in its fmalleft diameter. She had been in labour for above three days. As the child offered no refiftance for ufing the perforatorium, and as there was no poffibility of applying the forceps, the Cæfarean fection was thought neceffary, in order to deliver the woman. The firft incifion was made in the linea alba, to the extent of eight inches; the lofs of blood was inconfiderable. A correfponding incifion being now made in the uterus, which was hardly one line thick, the back of the child appeared; that was already putrid. The funiculus umbilicalis was cut through without much lofs of blood. The uterus contracted itfelf very faft, particularly round the placenta, which on that account was taken out with much difficulty. The application of the future caufed much pain to the patient. In the courfe of three months fhe was entirely cured, and all the fymptoms, during this period, had been very flight. On the feventh day after the operation, a piece of the omentum feparated, that was incarcerated in the lower part of the wound.

---

Another cafe of Cæfarean operation, which though performed under the moft happy appearances proved fatal, is related by *Dr. Klein,* of of Stuttgard. The pelvis of the woman was extremely diſtorted, and the child living. The inciſion in the abdomen was made on the right ſide, at a diſtance of two inches from the umbilicus, in an oblique direction towards the axis of the pelvis, becauſe the uterus, by contracting itſelf, became ſo inverted, that its internal ſurface was quite turned outwardly. The woman died four days after the operation. *Dr. Klein* ſtates, that of 116 caſes of the Cæſarean operation, which he found related, 90 were attended with ſucceſsful iſſue, whereas 26 proved mortal. It is however remarkable, that there are more fatal caſes mentioned in modern times, where the operation leſs frequently occurred, than from the year 1500 till 1769, when 6 mortal, and 76 ſucceſsful caſes are recorded. This, *Dr. Klein* ſeems inclined to aſcribe to the mode of treatment, which, according to him, ought to conſiſt of gentle purgatives, that do not weaken the conſtitution, and of cold ſtrengthening fomentations on the belly.

In addition to the above two caſes, Profeſſor *Loder*, of Jena, conſiders the Cæſarean operation by no means ſo difficult and dangerous as has been thought, as it is not attended with much pain, nor requires any particular art and dexterity. He is of opinion that, generally, a fatal termination enſued when it was undertaken too late, a circumſtance which may more eaſily occur when ſuch patient is attended by any great accoucheur, whoſe ſkill induces him to try other expedients which his art affords him, before he determines to undertake the Cæſarean operation. On the whole, Profeſſor *Loder* adviſes to make the inciſion in the linea alba.

---

*Dr. Wantzel* has made ſeveral remarks reſpecting *Mr. Home's* account of an orifice in the retina of the eye. (*Philoſophical Tranſactions*, 1798, P. II.), of which the reſults are the following. The conical tube in the vicinity of the optic nerve, which Mr. *Home* diſcovered in the eyes of a ſheep and a bullock; he likewiſe obſerved and found it indicated by ſome old anatomiſts, viz. *Brigg* ophthalmographia, ſive oculi ejuſque partium deſcriptio anatomica, Cantabrigiæ, 1676, p. 28. fig. 2. S. No. 11; *Winſlow*, Expoſition anatomique de la ſtructure du corps humain, Paris, 1732. Traité de la tete, No. 223; *Haller*, Comment. ad Boerhaavii prælect. eadem. v. iv. § 525. not. d. Element. Phyſiologiæ, corp. hum. T. v. p. 383; *Heiſter* anatomie avec des eſſais de phyſique, &c. Paris, 1735. p. 693. 1. It exiſts probably only in young animals, and is loſt when they grow older.—2. It is no lymphatic veſſel, but merely an eminence projected from the retina.—3. The canal which comes forth from this tube, as is to be obſerved in the calf's eye, and extends to the lens cryſtallina, going through another canal formed by the hyaloidea, may be traced in children, rabbits, hares, cats, &c.—4. This veſſel ſeems to be nothing but the arteria centralis.—5. The central artery does not, at leaſt in horſes and calves, enter into the eye by the ſame way with the vena centralis through the middle of the optic nerve, but on the outſide of it; and in the calf, through an aperture in the conic eminence of the retina.—6. *Dr. Wantzel* diſcovered, at ſome diſtance from the inſertion of the optic nerve, a ſimilar aperture in the retina of a horſe, as Mr. Soemmering had ſeen in the human eye. This aperture has likewiſe an oval form, and is incloſed by two veſſels; whereas the orifice in the human eye is ſurrounded with a thick margin.—7. The adheſion of the vitreous humour to the ſpot where the aperture is found, as obſerved by Mr. *Home* in men and a monkey, ſeems to ariſe from the central artery penetrating into the eye And thus we may conclude from analogy, that alſo in men and monkeys the central artery perforates the retina in a place different from that, whence the vena centralis penetrates into the eye, and that conſequently there ought to be in the retina, beſides the aperture which was already known to the ancients under the name of porus, another, viz. the orifice diſcovered by Mr. *Soemmering*.—8. The plait may have been occaſioned in adults by the preſſure and adheſion of the vitreous humour to that ſpot. It ſeems, however, not improbable, that alſo in men, at the firſt periods of life, the central artery penetrates a protuberance of the retina, which is loſt when the veſſel contracts itſelf by degrees.—9. The yellow ſpot is nothing but a phenomenon riſing only after death, and ſeems not to differ from any other ecchymoſis. Similar ſpots have been alſo obſerved by Dr. *Wantzel* in the lens cryſtallina of two horſes, that became blind from a previous inflammation of the eyes, by which blood was brought into the ſmalleſt and fineſt veſſels. The conſtant appearance of that yellow ſpot ſeems to depend on the veſſels which ſurround the above orifice.

---

*Dr. Golis*, phyſician to the inſtitution for infantile diſeaſes at Vienna, ſtates, in an account of the diſeaſes which occurred in this inſtitution, that among different affections to which the infantile age is expoſed, 7 children had been ſeized by what he ſtiles the *blue fever*, of which 6 recovered and 1 died. This morbid affection, which *Dr. Golis* did not find to be any where mentioned, attacks children, particularly from the age of 4 months to one year; without any evident cauſe, and without loſing their natural vivacity, or without ſhewing any ſenſe of pain, they begin to grow blue over the whole body; the natural heat decreaſes, the pulſe becomes ſlow and weak, a great laſſitude is perceived in all their motions, the eyes loſe their brightneſs and become languid, and are always fixed on one ſpot; the breath gets cold, the reſpiration quick and ſhort, is often imperceptible, and re-commences with a deep ſigh. On a ſudden they cry with a particular voice, bending themſelves, and their countenance expreſſes marks of the moſt violent pain. This laſts only a few minutes, but recurs again in a ſhort ſpace, being, at the ſame time, attended with a voracious appetite and an inſatiable thirſt. Their ſtools are white and hard; but a few hours before death, they become watery: the urine is without colour, and evacuated with preternatural copiouſneſs; the ſkin ſoft, but clammy and cold. In the progreſs of the diſeaſe, they grow weaker by degrees, they ceaſe to cry or give ſigns of pain, and die in a ſtupor, if medical aſſiſtance has been neglected in the beginning.

Mr.

Mr. Nemnich, of Hamburg, will ſhortly publiſh a complete Noſological Dictionary, in which the denominations of all the infirmities and diſeaſes of the human body, in the Latin, German, Engliſh, French, Italian, Spaniſh, and Portugueſe languages, are contained. It is to conſiſt of two parts, in the firſt of which the Latin terminology will be given, and in the ſecond, the dictionary of the above langugages, relating to diſeaſes, with a Latin explanation; price one ducat.

---

Mr. Girault praiſes the good effect of warm muriatic baths in arthritic complaints. He ſuffered himſelf a very obſtinate gout, againſt which, he uſed the warm ſaline baths at Pyrmont, during ſix weeks, with ſuch ſucceſs, that he felt himſelf greatly relieved after the fifth bath; and by degrees the ſwellings of the joints diſappeared, the limbs recovered their former activity, and the whole conſtitution was remarkably improved.

---

*Profeſſor Rooſe*, of Brunſwick, in a ſmall pamphlet, has made ſeveral remarks on the corpora lutea in the ovarium of women, on which particularly two modern authors, Dr *Haighton* and *Brugnoni*, have lately given their opinions, which in many points eſſentially differ from each other. The former agrees with *Regnar de Graaf* and *Haller*, that the preſence of the corpora lutea is an unqueſtionable proof of fecundation having taken place. The latter, however, thinks this opinion to be erroneous, maintaining that theſe bodies, which are remarked at the time of puberty, ought only to be conſidered as one of the ſeveral phenomena that indicate this period. *Profeſſor Rooſe* gives his reaſons, why he believes both phyſiologiſts to be equally wrong in their aſſertions; and he ſtates, 1. That it is an erroneous opinion to take the corpora lutea for *infallible* ſigns of previous conception or fecundation. 2. That it is improper to conſider them merely as marks of maturity, and the faculty of propagation in the other ſex. 3. That it is highly probable, theſe bodies might be formed, not only by fecundation, but alſo by a laſcivious imagination, and a topical irritation of the genitals.

---

*Dr. Thilow*, of Erfurt, has made ſeveral Galvanic experiments with ſaltpetre (nitrum), in order to ſhew, that it has a debilitating effect upon the nervous and muſcular fibre. On the crural nerve of a frog, nitre was applied, but without producing the leaſt convulſive motions, which, however, were immediately excited when it was touched with common ſalt. The lively motion of the heart of a frog was immediately ſtopped by a little nitre being ſcattered upon it.

---

The Pneumalkali, which was deſcribed by Dr. *Harhnemann*, as a new ſalt in *Crell's* Chemical Annals, from which we have given an extract in this Journal, and that ſold at the enormous price of 16 Louis d'ors a pound, is, according to the lateſt reſearches of Prof.

*Tromſdorf* and *Klaproth*, nothing elſe but mere borax. *Profeſſor Tromſdorf* very juſtly reproves Dr. Hahnemann for having attempted to impoſe upon the public with a degree of confidence that equals the impudence of the groſſeſt quack, as it is hardly probable Dr. H. ſhould have been ſo ignorant, as not to know its being really nothing but borax. How little, in future, is to be relied on the authority of Dr. H. we may hence eaſily conclude; and he certainly deſerves to loſe the credit of the public, which he gained by his former merit, but which he has of late ſo frequently miſuſed.

---

In *Hufeland's* Practical Journal, we find an ointment of manganeſe finely powdered, and ſuet, very much recommended in ſcabies, and it would be worth while to try it by farther experiments.

---

It is well known that the manner in which elephants couple has been hitherto unknown. By the diligent obſervations of *Mr. Corſe*, made in *Tiperah*, a province of Bengal, it is aſcertained that the elephants not only couple after the ſame way as ſtallions, but that, with a proper nouriſhment, they will propagate even in captivity. By the ſame experiments it is found, that the female elephant carries her young about twenty months.

---

To the Editors of the Medical and Physical Journal.

Gentlemen,

According to annual cuſtom I have tranſmitted to you the Prize Queſtions for the preſent year. I am, &c.

*Phyſical Society, Medical Theatre,* G. JOHNSON, Secretary.

*Guy's Hoſpital, April* 27, 1801.

Medical Question.

What is the nature of the diſeaſe called the Yellow Fever? and what is the beſt method of treating it?

Surgical Question.

What is the cauſe of the inflammation which ſucceeds wounds made into cavities, and what is the beſt mode of obviating the effects which they produce?

The ſucceſsful candidate for the prize, in anſwer to the queſtion (Are there any ſubſtances in Nature which act directly as ſedatives on the human body?) propoſed laſt year, was Mr. W. A Daingerfield, of Virginia, North America.

---

PRIZE QUESTION for 1801.

The Royal Medical Society of Edinburgh propoſe for the Subject of their Prize-Queſtion, a ſeries of experiments on the evolution of the Chick; and recommend the candidates for the prize, to direct their attention particularly to the following inquiries:

In what kinds of air can the procefs of evolution go on to maturity?

What changes in thefe airs accompany the procefs of evolution?

How much and what parts of the fhell may be covered with varnifh, without preventing the evolution of the Chick?

Has any kind of air the power to accelerate or to retard the procefs of evolution?

At what period of the evolution do the airs, unfit to fupport that procefs, prove moft fpeedily and moft irreparably injurious to the Chick?

Regulations relative to the Prize-Queftion.

1. That a Medal, or a Set of Books (at the option of the fuccefsful candidate) of five guineas value, be given annually to the author of the beft Differtation on an experimental fubject propofed by the Medical Society; for which all the members, Honorary, Extraordinary and Ordinary, fhall *alone* be invited as candidates.

2. The Differtations are to be written in Englifh or French, and to be delivered to the Secretary on or before the 31ft of December in the year fpecified, and the adjudication of the prize fhall take place in the laft week of February following.

3. To each Differtation fhall be prefixed a motto, and that motto is to be written on the outfide of a fealed packet, containing the name and addrefs of the author.—No Differtation can be received with the author's name affixed; and all Differtations, except the fuccefsful one, will be returned, if defired, with the fealed packet, unopened.

*Edinburgh, May* 19th, 1801.

---

### *On the Prevention of Contagious Fever among the Poor.*

We have great pleafure in laying before our readers an account of the meafures which have been adopted for forming an inftitution to prevent the progrefs of the contagious fever among the Poor of the metropolis.

The Society for bettering the condition of the Poor, ever attentive to the great object of their affociation, having requefted the opinion of the Faculty on the fubject, and having received a ftatement, figned by many of the moft refpectable phyficians to Hofpitals and Difpenfaries, appointed a meeting on the 1ft of May to take meafures for forming the Inftitution.

The attendance at this meeting was fuch as might have been expected. The Bifhop of Durham was requefted to take the chair, and the certificate from the Phyficians having been read,

Lord Sheffield faid, that after what the meeting had juft heard, it was not neceffary for him to detain them with many obfervations. He believed there could be but one opinion as to the importance of the fubject to which that paper alluded, and he therefore trufted that the refolutions which it was his intention to propofe would meet the unanimous approbation of the meeting. In the firft place, he wifhed to ftate the grounds on which would be founded the the meafures hereafter to be adopted; and he did not know that this could be done more effectually than by reciting the fubftance of the certificate. His Lordfhip, therefore, moved, as the firft refolution,

"That it appears to this meeting, by a certificate from the Phyficians of the hofpitals and difpenfaries in London, that the CONTAGIOUS MALIGNANT FEVER has been for fome time paft, and now is, prevalent in the metropolis;—and that it has been occafioned BY INDIVIDUAL INFECTION, which, with proper care, might have been immediately checked—or has been produced, or renewed, by the dwellings of the Poor not having been properly cleanfed and purified from contagion, after the fever has been prevalent in them: — That it alfo appears that this evil *(the injury and danger of which extend to every part of the metropolis)* might be prevented by cleanfing and purifying the clothes, furniture, and apartments of perfons attacked by this difeafe, — and by removing them from fituations where, if they remain, the infection of others is inevitable."

The motion being feconded, the refolution was unanimoufly agreed to. Lord Sheffield then moved the fecond refolution.

"That a Subfcription be immediately fet on foot, for the purpofe of forming an Inftitution for checking the progrefs of the CONTAGIOUS MALIGNANT FEVER in the metropolis, and for removing the caufes of Infection from the dwellings of the Poor, upon a plan fimilar to that which has been adopted with great fuccefs and effect at Manchefter."

Mr. Bernard feconded the motion, and faid, that he confidered the fuccefs of the meafure not as a matter of fpeculation or mere probability, but that it might be anticipated with perfect confidence and certainty. He had repeatedly witneffed the benefits produced by the Inftitution at Manchefter, and he trufted that the time had now arrived when the noble example of that populous town would be effectually followed by the metropolis. He then entered into a fhort account of the various advantages which had refulted from the eftablifhment of the Houfe of Recovery there, and concluded by repeating, that the meafure had his decided approbation.

This and the three following refolutions were then unanimoufly adopted.

"That a Committee of five perfons be appointed to prepare a Plan for the Inftitution, and to call a meeting of the Subfcribers, in order to lay the fame before them for their confideration.

"That the faid Committee do confift of the Bifhop of Durham, Dr. Garthfhore, Dr. Willan, W. Waddington, Efq. and Thomas Bernard, Efq.

"That the faid Committee be authorized to open accounts at fuch bankers in the metropolis as they fhall engage to receive fubfcriptions."

A fubfcription was then entered into, and amounted to nearly 600l. and the meeting was adjourned until the Committee fhall have have prepared a Plan for the Inſtitution. Among the firſt contributors were, the Ducheſs of Glouceſter, the Duke of Somerſet, the Earl of Pomfret, the Biſhops of London and Durham, Lord de Dunſtanville, Lord Carrington, Lord Sheffield, Mr. Bernard, Mr. Morton Pitt, and Mr. Wilberforce; and we are happy to hear that the liſt of Subſcribers has ſince received very conſiderable additions.

---

Dr. Dennison and Dr. Squire will begin early in next month a Summer Courſe of Lectures on the Theory and Practice of Midwifery, and the Diſeaſes of Women and Children.

